# No Association between *TNF-α* -308G/A Polymorphism and Idiopathic Recurrent Miscarriage: A Systematic Review with Meta-Analysis and Trial Sequential Analysis

**DOI:** 10.1371/journal.pone.0166892

**Published:** 2016-11-28

**Authors:** Jiashu Dong, Jinwan Li, Gechen Zhou, Zheng Peng, Jingjing Li, Shengzhang Lin, Haihua Liu, Chunlin Wu, Yujie Huang, Xiaolan Lv, Shengming Dai

**Affiliations:** 1 Department of Clinical Laboratory, the Fourth Affiliated Hospital of Guangxi Medical University, Liuzhou, Guangxi, China; 2 Department of Gynaecology and Obstetrics, the Fourth Affiliated Hospital of Guangxi Medical University, Liuzhou, Guangxi, China; 3 Department of Clinical Laboratory, Liuzhou Maternity and Child Health Care Hospital, Liuzhou, Guangxi, China; BC Children's Hospital, CANADA

## Abstract

**Background:**

Conflicting results were reported on the association between the *TNF-α* -308G/A polymorphism and idiopathic recurrent miscarriage (IRM). Though three meta-analyses have been conducted on this topic, the conclusions were contradictory, and the results may be unreliable as certain crucial conditions were neglected.

**Method:**

A complete search was conducted in PubMed, Cochrane Library, and Embase, other sources like Google Scholar, ClinicalTrial.gov and reference lists of relevant articles were also retrieved. All candidate articles were accessed and screened using specific inclusion and exclusion criteria. Statistical analyses were performed on data extracted from eligible studies using the STATA 12.0 software and the TSA 0.9 beta software.

**Results:**

Eventually, 12 case-control studies from 11 publications (with 1,807 cases and 2,012 controls) were included in this meta-analysis, and no evidence of any significant association was found in the overall analyses between the *TNF-α* -308G/A polymorphism and IRM risk. However, significant association was shown in Asian population (four studies from three publications) in the dominant model (AA + GA vs. GG), the allelic model (A vs. G), and the heterozygote model (GA vs. GG).

**Conclusions:**

*TNF-α* -308G/A polymorphism is not associated with IRM risk. Though significant association was found in Asian population, the result needs further confirmation from more studies.

## Introduction

Spontaneous miscarriage, which afflicts 10% to 20% of pregnant women [1–4], is a distressing experience and a common complication in early pregnancy [5, 6]. To make matters worse, 1% to 5% of women will suffer two or more consecutive, unexplained pregnancy losses with the same partner prior to the 20th week of gestation [7, 8]. This is called idiopathic recurrent miscarriage (IRM) [9]. IRM is related to a variety of causes [10], among which the disturbance of the maternal homeostatic balance between the Th1 and Th2 cytokine system, is best studied [11–13]. This balance is maintained by a series of cytokines [14]. It has been reported that Th_1_ cytokines are detrimental and associated with IRM, whereas Th_2_ cytokines are not [15, 16].

As a pro-inflammatory Th1 cytokine, tumor necrosis factor alpha (TNF-α) is mapped in chromosome 6p21.3 and mainly secreted by mono-nuclear phagocytes, lymphocytes, and natural killer (NK) cells [17]. Some researches have demonstrated that TNF-α is implicated in the development of IRM [18–21], possibly by inducing the apoptosis of trophoblasts and promoting the expression of apoptotic genes in the human fetal membrane [22, 23]. Moreover, the production of TNF-α is mainly controlled by genes, whereas mutations of these genes could result in changes of TNF-α level, especially in the promoter region [24, 25]. Therefore, polymorphisms in this region may be associated with IRM risk. And a bunch of studies have been performed to evaluate the association between TNF-α promoter polymorphisms and IRM risk [26–50].

Among all the genetic variants in this region, *TNF-α* -308G/A (rs1800629) is most studied [28–50]. However, the results of these studies are inconsistent and often conflicting. Although one meta-analysis of 7 studies in 2009 [48], another one of 12 studies in 2012 [49], and the third one of 10 studies in 2016 [50] have been conducted, their conclusions were conflicting and unreliable due to the inclusion of studies deviating significantly from Hardy-Weinberg equilibrium (HWE) [39, 40], and studies without sufficient data to calculate HWE [44–47]. Meanwhile, a missing study [32] in the previous meta-analyses and a couple of new studies with different results [38, 41, 42] were found by us during the investigation. Therefore, we conducted this study to obtain more concrete and conclusive conclusions concerning the correlation between the *TNF-α* -308G/A polymorphism and IRM through a comprehensive and robust meta-analysis.

## Materials and Methods

The present meta-analysis was conducted in accordance with the PRISMA (Preferred Reporting Items for Systematic Reviews and Meta-Analyses) guidance (S1 Table). No review protocol was registered for this study.

### Identification of eligible trials

Relevant articles were identified by a comprehensive search of the following electronic databases through July 2016: PubMed, Cochrane Library, Embase. The search terms included the synonyms of miscarriage, tumor necrosis factor and polymorphism (S1 File). The SNP number (rs1800629) was also searched in combination with the synonyms of miscarriage. In addition, Google Scholar, ClinicalTrial.gov and reference lists of relevant articles were also screened by two authors independently to collect the randomized controlled trials (RCTs) published.

### Inclusion and exclusion criteria

For all the relevant literature, the following inclusion criteria were adopted: 1) case-control designed studies or retrospective cohort studies with clear inclusion criteria; 2) data on allele and genotype frequencies provided; and 3) information on DNA genotyping method and characteristics of cases and controls included. Studies without genotype data or with duplicate data were excluded. Letters, case reports, editorials, review articles, conference abstracts, and animal studies were also excluded. Eligible studies were selected by the same two authors independently by screening the title, abstract, and full article based on the above criteria. Disputes were solved by consultation.

### Data extraction

From all eligible studies, the following data were extracted: last name of the first author, publication date, country, ethnicity, mean age and source of the cases and controls, total sample size, genotype frequencies, and genotyping method. For each study, the HWE of the control group was computed from the genotype frequencies extracted above, and studies with *p* <0.05 were considered as significantly deviating from the HWE and would be excluded from this meta-analysis. If a study had subgroups, each subgroup would be listed as a separate study. Two authors completed the whole process independently. If differences existed, data would be rechecked independently by the two authors. Further discrepancies would be referred to a third author. To obtain necessary missing data, authors were contacted via e-mail.

### Quality assessment

The quality of each study was assessed using the assessment scale adapted from Peng et al. [51] for the present meta-analysis (Table 1). Each study was scored and labelled as either low quality (score ≤6) or high quality (score >6) based on items such as the definition of IRM adopted, representativeness of controls, description of genotyping method, mean age of cases, and total sample size. The quality assessment was performed by two authors independently, and disagreements were settled by consultation.

**Table 1:** Scale for quality assessment of studies included.

Criteria^a^	Score
IRM definition	
≥3 consecutive miscarriages	1
≥2 consecutive miscarriages	0
Representativeness of controls	
Population-based (PB)	2
Hospital-based(HB)	1
Not described	0
Genotyping method	
Described	1
Not described	0
Mean age of cases	
≤ 35	3
≤ 40	1
> 40 or not described	0
Total sample size	
≥ 500	3
≥ 200	2
≥ 100	1
< 100	0

^a^ These criteria are unfit for studies inconsistent with HWE.

### Statistical analysis

Based on the genotype frequencies of cases and controls in each study, we conducted a series of overall meta-analyses using the following five genetic models: the homozygote model (AA vs. GG), the heterozygote model (GA vs. GG), the recessive model (AA vs. GA + GG), the dominant model (AA + GA vs. GG), and the allelic model (A vs. G). Then, using the odd ratio (OR) and confidence interval (CI) produced, we evaluated the association between the above genetic models and IRM risk. The overall significance of the association was calculated by a paired z-test, and a *p* value < 0.05 was considered significant. Heterogeneity among studies was computed by the Q statistic and the *I*^*2*^ statistic. For each study, either the fixed-effects model or the random-effects model was used, based on the *P*_*Q*_ value. If the *P*_*Q*_ value was >0.1, the former was adopted; otherwise, the latter was chosen. To investigate the influence of primary characteristics and explore the source of heterogeneity, we conducted a series of subgroup analyses. In addition, we conducted a sensitive analysis to test the stability of the overall results by sequentially taking out one study each time, a cumulative meta-analysis to portray the shift of the association over time by adding studies one by one based on publication date, and a trial sequential analysis (TSA) to minimize the risk of type I errors. Furthermore, we performed Galbraith plot to facilitate the examination of heterogeneous studies. For the evaluation of publication bias, Egger’s regression test were performed. Funnel plots and Egger’s publication-bias plots were also generated in the process. All analyses were conducted using STATA software version 12.0 and TSA software version 0.9 beta. Two-tailed *p* values <0.05 were considered as statistically significant.

## Results

### Literature selection

**Fig 1:**
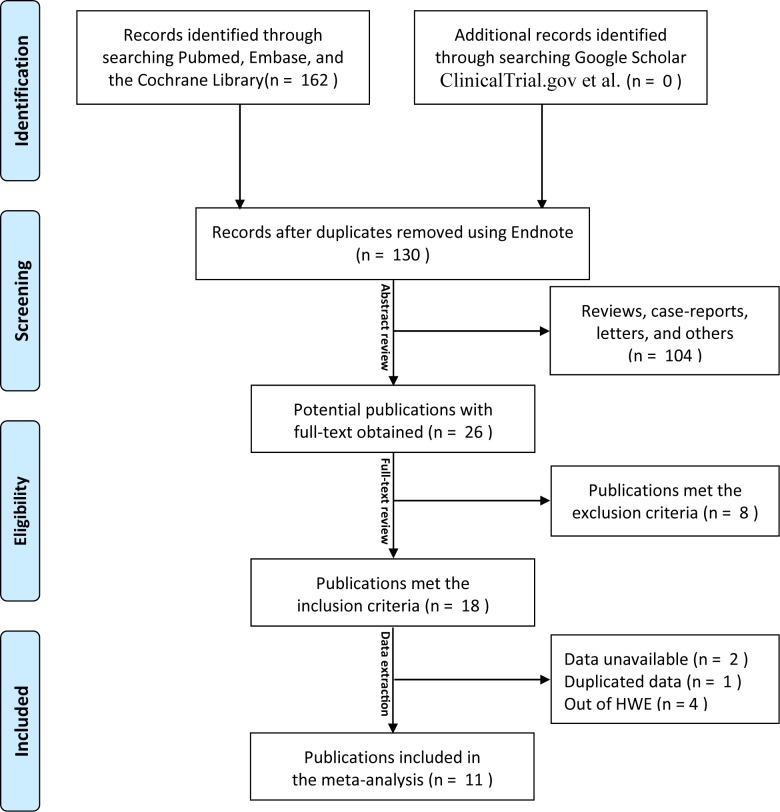
Flow diagram of search and selection for studies.

The initial search generated 162 relevant records, of which 32 were duplicates. After reviewing the abstracts of the remaining 130 records, 104 records were ruled out as irrelevant articles, reviews, letters or case-reports. The full texts of the left 26 potential publications were obtained and reviewed. Among them, three publications without sufficient data [44–47], one with duplicated data [43], and four out of HWE [39–42] were excluded. Eventually, 12 studies from 11 publications [28–38] were included in the meta-analysis. Fig 1 illustrates the process of search and selection. S2 File details the excluded articles and the reasons for their exclusion, as well as the original data obtained from the author via e-mail. No genome-wide association studies (GWAS) was found on this topic.

### Characteristics of included studies

Table 2 summarizes the primary characteristics of the 12 studies finally included in the analysis. As for ethnicity, there are four studies from three publications [34, 36, 38] conducted in Asians, whereas eight studies [28–33, 35, 37] in Caucasians. All articles are in English, except one in Spanish with English abstract [32].

**Table 2:** Primary characteristics of the 12 studies included in the meta-analysis.

Study	Study location	Ethnicity	Sourceofcontrol	Age		Definition ofIRM	Samples size			Genotypingmethod	Case		Control		Qualityscore
											Genotype frequency	HWE*P*	Genotype frequency	HWE*P*	
				Case	Control		Case	Control	Total		AA/AG/GG		AA/AG/GG		
Babbage,2001 [28]	UK	Caucasian	HB	21–45	30–58	≥3	43	73	116	PCR-ASA	1/12/30	0.876	3/14/56	0.107	7
Reid,2001 [29]	UK	Caucasian	HB	-	-	≥2	17	43	60	PCR-RFLP	2/6/9	0.536	1/13/29	0.744	2
Pietrowski,2004 [30]	Germany	Caucasian	PB	33	58	≥3	168	212	380	PCR	2/33/133	0.977	4/41/167	0.432	7
Kamali,2005 [31]	Iran	Caucasian	PB	18–42	-	≥3	131	143	274	PCR-ASO	0/14/117	0.518	0/21/122	0.343	6
Quintero,2006^a^ [32]	Mexico	Caucasian	PB	-	-	≥3	122	214	336	PCR	1/8/113	0.067	2/30/182	0.544	5
Zammiti,2009 [33]	Tunisia	Caucasian	PB	29	29	≥3	372	274	646	PCR-RFLP	14/39/319	0	5/47/222	0.187	10
Liu,2010 [34]	China	Asian	HB	30	29	≥2	132	152	284	PCR	0/22/110	0.296	1/13/138	0.276	6
Palmirotta,2010 [35]	Italy	Caucasian	PB	37	38	≥2	100	100	200	PCR	0/13/87	0.487	3/21/76	0.313	5
Gupta,2012 [36]	India	Asian	PB	-	-	≥3	300	500	800	PCR-RFLP	9/62/229	0.067	5/70/425	0.274	7
Alkhuriji,2013 [37]	Saudi Arabia	Caucasian	HB	34	-	≥3	65	65	130	PCR-SSP	8/24/33	0.282	4/14/47	0.059	7
Lee1,2013 [38]	South Korea	Asian	PB	33	33	≥2	187	236	423	PCR-RFLP	1/21/165	0.711	2/21/213	0.083	8
Lee2,2013 [38]	South Korea	Asian	PB	33	33	≥3	170	236	406	PCR-RFLP	1/15/154	0.353	2/21/213	0.083	9

PCR-RFLP, polymerase chain reaction-restriction fragment length polymorphism; PCR-ASA, polymerase chain reaction-allele specific amplification; PCR-ASO, polymerase chain reaction-allele specific oligonucleotide; PCR-SSP, polymerase chain reaction-sequence specific primers; HWE, Hardy-Weinberg equilibrium; PB, population based; HB, hospital based

^a^, this article is in Spanish with English abstract.

### Meta-analysis results

**Fig 2:**
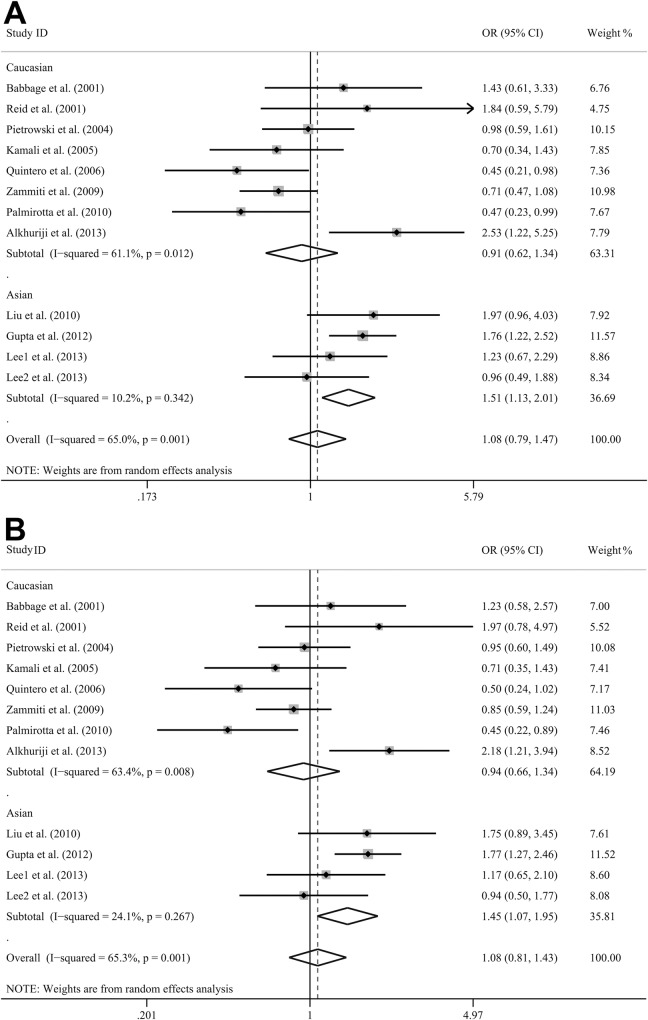
Forest plots for the association between *TNF-α* -308G/A Polymorphism and IRM risk classified by ethnicity in dominant model (A) and allelic model (B).

Table 3 presents the primary results of all five genetic models in this comprehensive meta-analysis. No significant association was detected in the overall meta-analysis. As there were one studies [31] with zero AA phenotype in both cases and controls, the overall meta-analysis was performed with only 11 studies in the homozygous model (AA vs. GG) and the recessive model (AA vs. GA + GG). The subgroup meta-analyses showed significant associations in Asian subjects in the dominant model, the allele model, and the heterozygote model between *TNF-α* -308G/A and IRM risk (Fig 2). Similar results were found in the hospital-based-control group and the total-sample-size <150 group. No significant associations were found in any of the subgroups classified by the definition of IRM, the score or the Galbraith plot.

**Table 3:** Primary results of overall meta-analyses and subgroup analyses.

Comparison	Group	No. of studies	Test of association			Effect model	Test of heterogeneity	
			OR	95%CI	*P*_*OR*_		*I*^*2*^ (%)	*P*_*Q*_
**-308G/A (rs1800629)**		**12**						
**AA vs.GG**	**Overall**	**11**^**a**^	**1.49**	**(0.93,2.40)**	**0.098**	**fixed**	**4.7**	**0.399**
**AG vs.GG**	**Overall**	**12**	**1.06**	**(0.77,1.46)**	**0.716**	**random**	**63.7**	**0.001**
**AA vs.AG + GG**	**Overall**	**11**^**a**^	**1.27**	**(0.77,2.10)**	**0.344**	**fixed**	**0**	**0.692**
**AA + AG vs.GG**	**Overall**	**12**	**1.08**	**(0.79,1.47)**	**0.632**	**random**	**65**	**0.001**
Ethnicity	Asian	4	1.52	**(1.17,1.98)**^**s**^	0.002	fixed	10.2	0.342
	Caucasian	8	0.91	(0.62,1.34)	0.639	random	61.1	0.012
Definition of IRM	≥3 miscarriages	8	1.05	(0.72,1.52)	0.818	random	69.5	0.002
	≥2 miscarriages	4	1.17	(0.61,2.24)	0.637	random	64.2	0.039
Source of control	HB	4	1.97	**(1.31,2.95)**^**s**^	0.001	fixed	0	0.796
	PB	8	0.87	(0.61,1.23)	0.436	random	67	0.003
Total sample size	<150	3	1.96	**(1.20,3.22)**^**s**^	0.008	fixed	0	0.600
	>150	9	0.94	(0.67,1.32)	0.725	random	67.5	0.002
Score	≤6	5	0.85	(0.46,1.59)	0.612	random	67.4	0.016
	>6	7	1.23	(0.88,1.72)	0.229	random	61.4	0.016
Galbraith plot	insiders^b^	8	1.03	(0.81,1.31)	0.834	fixed	33.9	0.158
	outliers^c^	4	1.11	(0.56,2.20)	0.760	random	85.3	0
**A vs. G**	**Overall**	**12**	**1.08**	**(0.82,1.43)**	**0.595**	**random**	**65.3**	**0.001**
Ethnicity	Asian	4	1.49	**(1.17,1.90)**^**s**^	0.001	fixed	24.1	0.267
	Caucasian	8	0.94	(0.66,1.44)	0.728	random	63.4	0.008
Definition of IRM	≥3 miscarriages	8	1.06	(0.77,1.48)	0.714	random	67.9	0.003
	≥2 miscarriages	4	1.13	(0.60,2.15)	0.706	random	69.8	0.019
Source of control	HB	4	1.78	**(1.25,2.53)**^**s**^	0.001	fixed	0	0.687
	PB	8	0.90	(0.64,1.24)	0.485	random	68.8	0.002
Total sample size	<150	3	1.79	**(1.19,2.70)**^**s**^	0.005	fixed	0	0.478
	>150	9	0.95	(0.69,1.30)	0.743	random	67.7	0.002
Score	≤6	5	0.86	(0.47,1.57)	0.631	random	69.9	0.01
	>6	7	1.23	(0.92,1.64)	0.166	random	57.3	0.029
Galbraith plot	insiders^b^	8	1.01	(0.81,1.26)	0.947	fixed	29.1	0.196
	outliers^c^	4	1.13	(0.62,2.07)	0.688	random	85.2	0

CI, confidence interval; OR, odds ratio; IRM, idiopathic recurrent miscarriage; ^a^, one studies [31] are not analyzed due to 0 AA genotype in both case and control group; Of the 12 studies included in this meta-analysis, 8 studies from 7 articles [28–32, 34, 38] are insiders^b^, 4 studies [33, 35–37] are outliners^c^ in Galbraith plot (S1 Fig); ^s^, significant results.

### Publication bias

**Fig 3:**
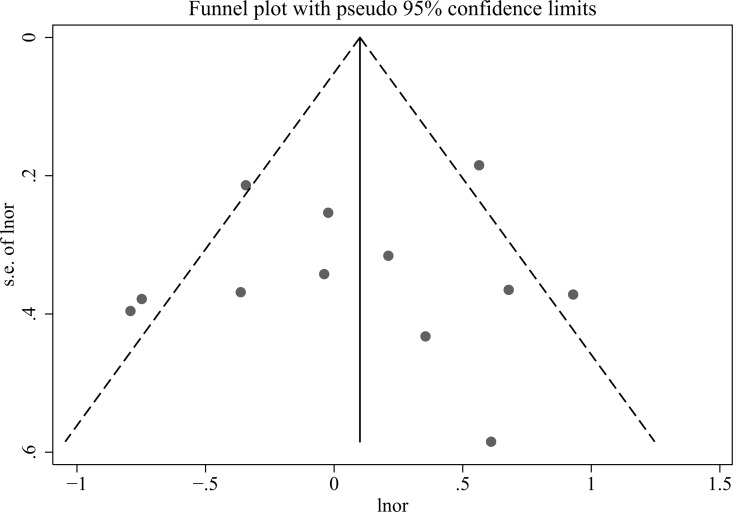
Funnel plot of the 12 studies included in this meta-analysis.

The results of Egger’s test confirmed that no significant publication bias existed in our meta-analysis (S3 File). Furthermore, Funnel plot (Fig 3), Egger’s publication-bias plot (S3 File) of the 12 studies demonstrated no sign of significant publication bias.

### Heterogeneity analysis

*I*^*2*^ and *P*_*Q*_ values showed significant heterogeneity among the 12 studies in 3 genetic models (AG vs. GG; AA + GA vs. GG; A vs. G), whereas 2 genetic models (AA vs. GG; AA vs. AG + GG) with 11 studies available for analysis demonstrated little heterogeneity (Table 3). Galbraith plot (S1 Fig) of the included 12 studies confirmed the existence of significant heterogeneity and illustrated 4 studies [33, 35–37] were the outliners. All the subgroup analyses showed a decline in heterogeneity in at least one subgroup, except the subgroups classified by the definition of IRM (Table 3).

### Sensitivity analysis

In the sensitivity analysis, the pooled standardized mean difference (SMD) and 95% CIs were not significantly affected, no matter which study was taken out. (S2 Fig).

### Cumulative meta-analysis

In the cumulative meta-analysis, no significant association between *TNF-α* -308G/A and IRM ever appeared over time (S3 Fig).

### Trial sequential analysis

**Fig 4:**
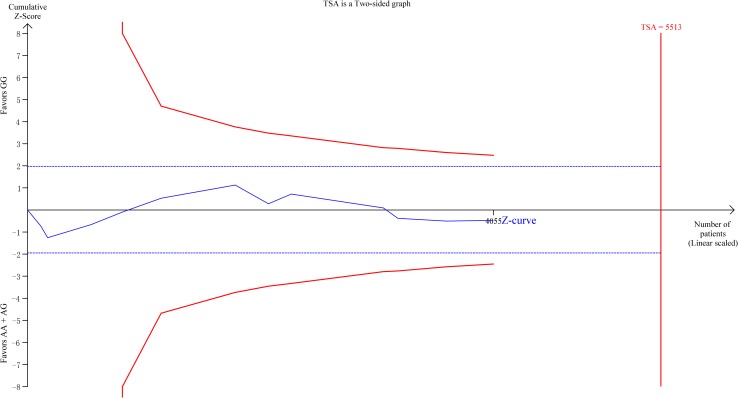
Trial sequential analysis of the studies included. A two-sided graph is plotted by TSA where the blue etched lines represent conventional significance boundaries, the blue line indicates the cumulative Z-score, and the red lines shows the α -spending boundary and the required information size.

Repeated tests for significance upon new trials by meta-analyses may incur type I error [52]. To evaluate and minimize it, TSA was employed using software version 0.9 beta [53]. TSA combines traditional meta-analysis with information size calculation, and methods to adjust the significance according to the quantified strength of evidence and the impact of multiplicity for the repeated tests on accumulating trial data. In the present analysis, TSA was performed in dominant model with a two-tailed alpha of 0.05, beta of 0.20, and a relative risk increase of 10%. And a constant value correction of 0.5 in the no event trials was applied. The result of TSA demonstrates that neither the traditional significance boundaries nor the α-spending boundaries is crossed by the cumulative z-curve (dominant model) (Fig 4).

## Discussion

The present meta-analysis enrolled 12 studies from 11 publications with 1,807 cases and 2,012 controls to assess the correlation between *TNF-α* -308G/A polymorphism and IRM risk. In addition, this meta-analysis presented the first cumulative meta-analysis and the first trial sequential analysis on this topic. In the cumulative meta-analysis, we found no trend of association, and more stable CIs with the accumulation of studies based on publication dates (S3 Fig). The trial sequential analysis shows a parallel cumulative z-curve to both the conventional boundaries and the α-spending boundaries (Fig 4), which indicates and confirms the inexistence of association between *TNF-α* -308G/A and IRM (S4 Fig). Sensitivity analysis also demonstrated that the overall results were reliable and robust, with no single influential study. All in all, the overall analyses found no significant associations in all five genetic models, which is quite opposite to the results of two recent meta-analyses in 2012 and 2016 [49, 50], whereas consistent with the meta-analysis in 2009 [48] and two reviews about the effect of polymorphisms of TNF-α[54, 55].

Since the main difference between the present meta-analysis and the previous meta-analyses [48–50], primarily lies in the exclusion and inclusion of studies without sufficient data to calculate HWE [44–47], and studies deviating from HWE [39–41] (Table 4). After looking into the previous meta-analyses with great care, we noticed that some data could not be found in the original manuscripts [31, 44–46] were listed out and analyzed in the meta-analyses in 2009 [48] and 2012 [49], and some studies without sufficient data to calculate HWE [28, 31, 45, 46] were included and analyzed in the meta-analysis in 2016 [50] (Table 4). The possibility exists that the suspicious data in the meta-analysis in 2012 may be obtained from authors directly. Hence, an additional meta-analysis was conducted with these suspected data, and the primary results remain unchanged (S4 File). Therefore, we may conclude that the associations found in previous meta-analyses were skewed due to the studies inconsistent with HWE. After all, departure from HWE can indicate systematic errors in genotyping, and data generated under this condition were unreliable and may significantly affects the conclusions of meta-analysis, which is the reason why HWE was ranked as an essential and routine item of the scrutinizing procedure in population-based genetic association meta-analyses (S2 Table, Item 9) [56].

**Table 4:** Primary differences between previous meta-analyses and the present meta-analysis on the association of *TNF-α* -308G/A Polymorphism and IRM.

Author/Year	Country	Original data (genotype frequency)		HWE	Definition of IRM	Data in the Meta-analysis of 2009[47]		Data in the Meta-analysis of 2012[48]		Data in the Meta-analysis of 2016[49]		Data in the present meta-analysis	
		Case^a^	Control^a^			Case^a^	Control^a^	Case^a^	Control^a^	Case^a^	Control^a^	Case^a^	Control^a^
Babbage, 2001[28]	UK	1/12/30	3/14/56	Y	≥3	1/12/30	3/14/56	1/12/30	3/14/56	13/30	17/56	1/12/30	3/14/56
Baxter, 2001[44]	UK	-	-	-	≥3	*25/51*^*b*^	*44/94*^*b*^	*3/22/51*^*b*^	*5/40/93*^*b*^	-	-	-	-
Reid, 2001[29]	UK	2/6/9	1/13/29	Y	≥2	2/6/9	1/13/29	2/6/9	1/13/29	excluded^e^	excluded^e^	2/6/9	1/13/29
Daher, 2003[45]	Brazil	12/36	19/89	-	≥3	12/36	19/89	1/11/36 ^*b*^	1/18/89 ^*b*^	12/36	19/89	excluded^d^	excluded^d^
Pietrowski, 2004[30]	Germany	2/33/133	4/41/167	Y	≥3	2/33/133	4/41/167	2/33/133	4/41/167	2/33/133	4/41/167	2/33/133	4/41/167
Prigoshin, 2004[46]	Argentina	6/35	5/49	-	≥3	6/35	5/49	0/6/35 ^*b*^	0/5/49 ^*b*^	6/35	5/49	excluded^d^	excluded^d^
Kamali, 2005[31]	Iran	14/117	21/122	-/Y ^c^	≥3	14/117	21/122	0/14/117 ^*b*^	0/21/122 ^*b*^	14/117	21/122	0/14/117 ^c^	0/21/122 ^c^
Quintero, 2006[32]	Mexico	1/8/113	2/30/182	Y	≥3	missing	missing	missing	missing	missing	missing	1/8/113	2/30/182
Zammiti, 2009[33]	Tunisia	14/39/319	5/47/222	Y	≥3			14/39/319	5/47/222	14/39/319	5/47/222	14/39/319	5/47/222
Finan, 2010[39]	Bahrain	8/32/164	4/32/212	N	≥3			8/32/164	4/32/212	8/32/164	4/32/212	excluded^d^	excluded^d^
Liu, 2010[34]	China	0/22/110	1/13/138	Y	≥2			0/22/110	1/13/138	excluded^e^	excluded^e^	0/22/110	1/13/138
Palmirotta, 2010[35]	Italy	0/13/87	3/21/76	Y	≥2			0/13/87	3/21/76	excluded^e^	excluded^e^	0/13/87	3/21/76
Kaur, 2011[40]	India	5/6/39	2/7/41	N	≥3					5/6/39	2/7/41	excluded^d^	excluded^d^
Gupta, 2012[36]	India	9/62/229	5/70/425	Y	≥3					9/62/229	5/70/425	9/62/229	5/70/425
Alkhuriji, 2013[37]	Saudi Arabia	8/24/33	4/14/47	Y	≥3					8/24/33	4/14/47	8/24/33	4/14/47
Lee1, 2013[38]	South Korea	1/21/165	2/21/213	Y	≥2					excluded^e^	excluded^e^	1/21/165	2/21/213
Lee2, 2013[38]	South Korea	1/15/154	2/21/213	Y	≥3					missing	Missing	1/15/154	2/21/213
Bompeixe, 2013[47]	Brazil	16/45	16/59	-	≥2					excluded^e^	excluded^e^	excluded^d^	excluded^d^
Liu, 2015[41]	China	35/105/144	18/61/205	N	≥3							excluded^d^	excluded^d^
Sudhir, 2016[42]	India	5/34/76	6/18/87	N	≥2							excluded^d^	excluded^d^

HWE, Hardy-Weinberg equilibrium; -, data that could not be extracted from the original publications or calculated from genotype frequencies extracted; data in italic and bold, dubious and conflicting data found in two different meta-analyses

^a^, data of genotype frequency are sequenced in the order of AA/AG/GG, OR AA+AG/GG

^b^, dubious data found in the meta-analysis in 2012, but could not be found in the original publications

^c^, data obtained via e-mail from author

^d^, studies excluded due to deviating from HWE or insufficient data to calculate HWE

^e^, studies excluded because of different definition of IRM (In these studies, the authors adopted the definition of IRM as ≥2 consecutive spontaneous miscarriages, instead of ≥3 consecutive spontaneous miscarriages).

What is more, we conducted several stratified analyses to trace the possible sources of the heterogeneity and found several points noteworthy. First, as for the definition of IRM, no significant difference was shown between the ≥2 miscarriages group and the ≥3 miscarriages group (Table 3), indicating that the different definition of IRM is not the possible cause of heterogeneity. Similar result was also found by Lee et al [38], who performed stratified analysis according to the number of consecutive spontaneous abortions in the study. In practice, most clinicians generally work to the less rigorous ≥2 miscarriages definition, probably because patients will be extremely worried under this condition and it is doctor’s responsibility to address their problems. Second, in addition to the significant association, a lower heterogeneity was also seen in the Asian populations compared to the Caucasian populations. On one hand, it is possible that the association between *TNF-α* -308G/A polymorphism and IRM risk is of ethnic specificity. However, this result should be interpreted with care, since the association found in the Asian population was supported by four studies from only three publications, which is far from sufficient. On the other hand, the low heterogeneity may be due to the limited studies themselves. Third, eight insiders and four outliers discovered in Galbraith plot were classified and analyzed, and a remarkable decrease of heterogeneity were demonstrated among the 8 insiders in the dominant model (*I*^*2*^ 33.9) and the allelic model (*I*^*2*^ 29.1), indicating the four outliners may be the cause of the heterogeneity (Table 3). Fourth, there are signs of possible sample-selection bias. As mentioned above, one study [34] with zero AA genotype frequency in both cases and controls, is not included in the overall meta-analyses of both the homozygous model (AA vs. GG) and the recessive model (AA vs. GA + GG). And a significant decrease in heterogeneity is demonstrated in both model (with *I*^*2*^ 4.7 and 0, respectively). One possible explanation is that either the cases or the controls in this study are not representative. Another indication is that the heterogeneity of the total-sample-size >150 group was significantly higher than that of its counterpart group.

More importantly, during this meta-analysis, we summarized several meaningful points that may be helpful to further studies. First, the inclusion and exclusion criteria must be specific and comprehensive and should be strictly carried out. Otherwise, confounding factors will inevitably be mixed in. Second, more studies on Asian populations are needed. Third, more attention should be paid to the selection of the control group, which is always neglected and carried out without strictly following criteria. Four studies [39–42] deviating from HWE and one study with 0 AA genotype frequency in both cases and controls [31], are good representations of this issue.

There are some limitations in the present meta-analysis, which should be noted. First, misclassification bias and selection bias may be incurred due to unified diagnostic criteria of IRM and various sources of controls. Second, we failed to evaluate the gene-gene and gene-environment associations due to lack of the original data. Third, heterogeneity in several subgroups remains high in the subgroup analyses. Last but not least, the limited number of studies in Asian population may restrict the statistical power of the association.

In summary, no association between the *TNF-α* -308G/A promoter polymorphism and IRM was found in the present meta-analysis. The correlation found in Asian population needs confirmation from more studies.

## Conclusions

The present meta-analysis demonstrated no association between *TNF-α* -308G/A polymorphism and IRM risk, and the association found in the previous meta-analyses may result from the inclusion of studies inconsistent with HWE. Significant association demonstrated in Asian subjects in the subgroup analyses, should be interpreted with caution due to limited studies. Further rigorously-designed large-scale studies on Asian population are needed to confirm this conclusion.

## Supporting Information

S1 Fig Galbraith plot. (TIF)

S2 Fig Sensitivity analysis plot. (TIF)

S3 Fig Cumulative meta-analysis plot. (TIF)

S4 Fig Trial sequential analysis result summary (dominant model). (TIF)

S1 File Search strategy and search results. (PDF)

S2 File Data obtained via email & Excluded studies categorized by reasons. (PDF)

S3 File Results of Publication Bias Test & Figures. (PDF)

S4 File Additional analyses with dubious data. (PDF)

S5 File Additional analyses with the Chinese article. (PDF)

S1 Table PRISMA Checklist. (DOC)

S2 Table Meta-analysis on genetic association studies form. (DOCX)

S3 Table Inclusion criteria of all the studies included. (DOCX)
