# Supplementary figures and images for: No Association between TNF-α -308G/A Polymorphism and Idiopathic Recurrent Miscarriage: A Systematic Review with Meta-Analysis and Trial Sequential Analysis
Source: PLoS One. 2016 Nov 28;11(11):e0166892. doi: 10.1371/journal.pone.0166892 (PMC5125640; doi:10.1371/journal.pone.0166892)

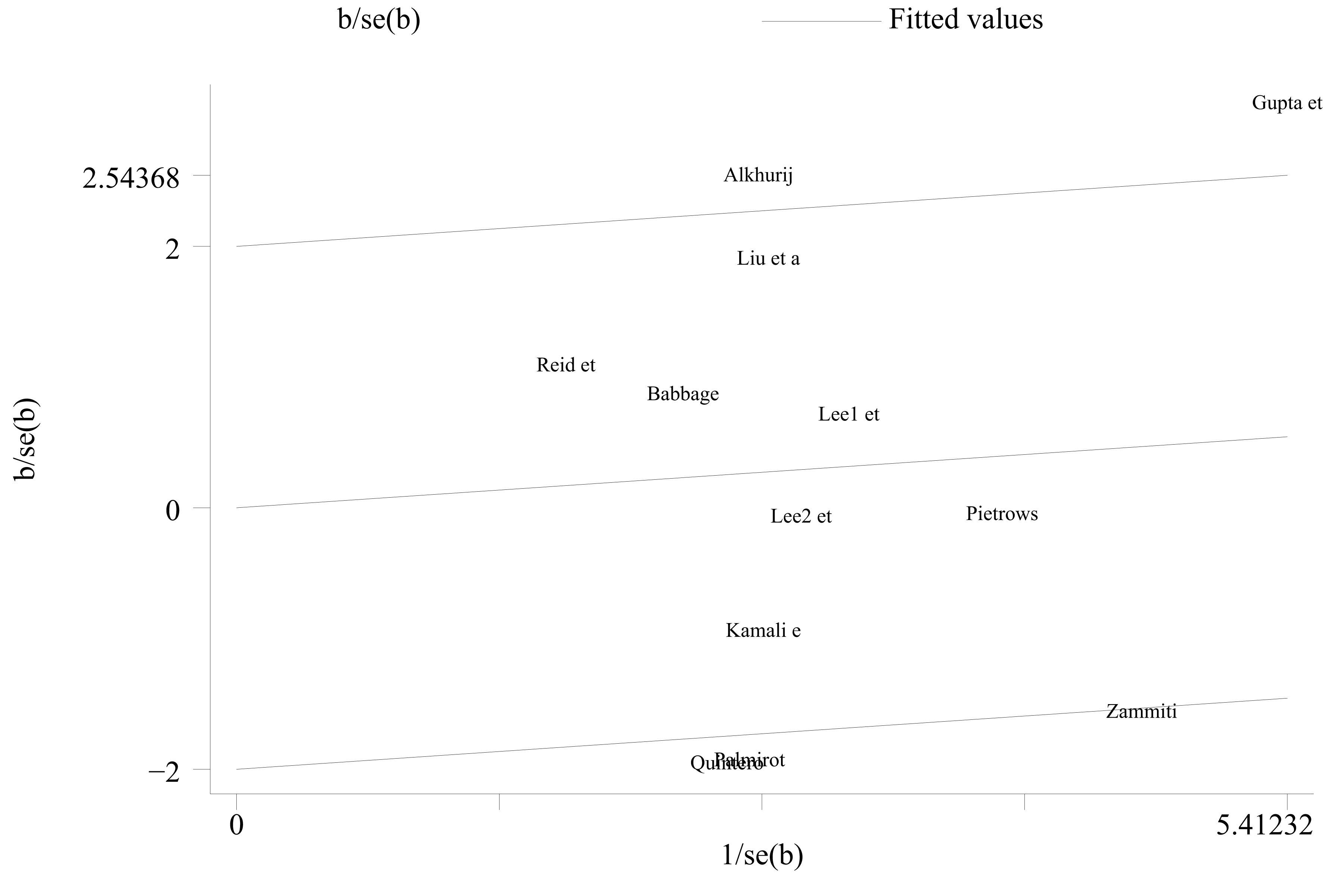

Supplement: S1 Fig — (TIF) [file pone.0166892.s001.tif]

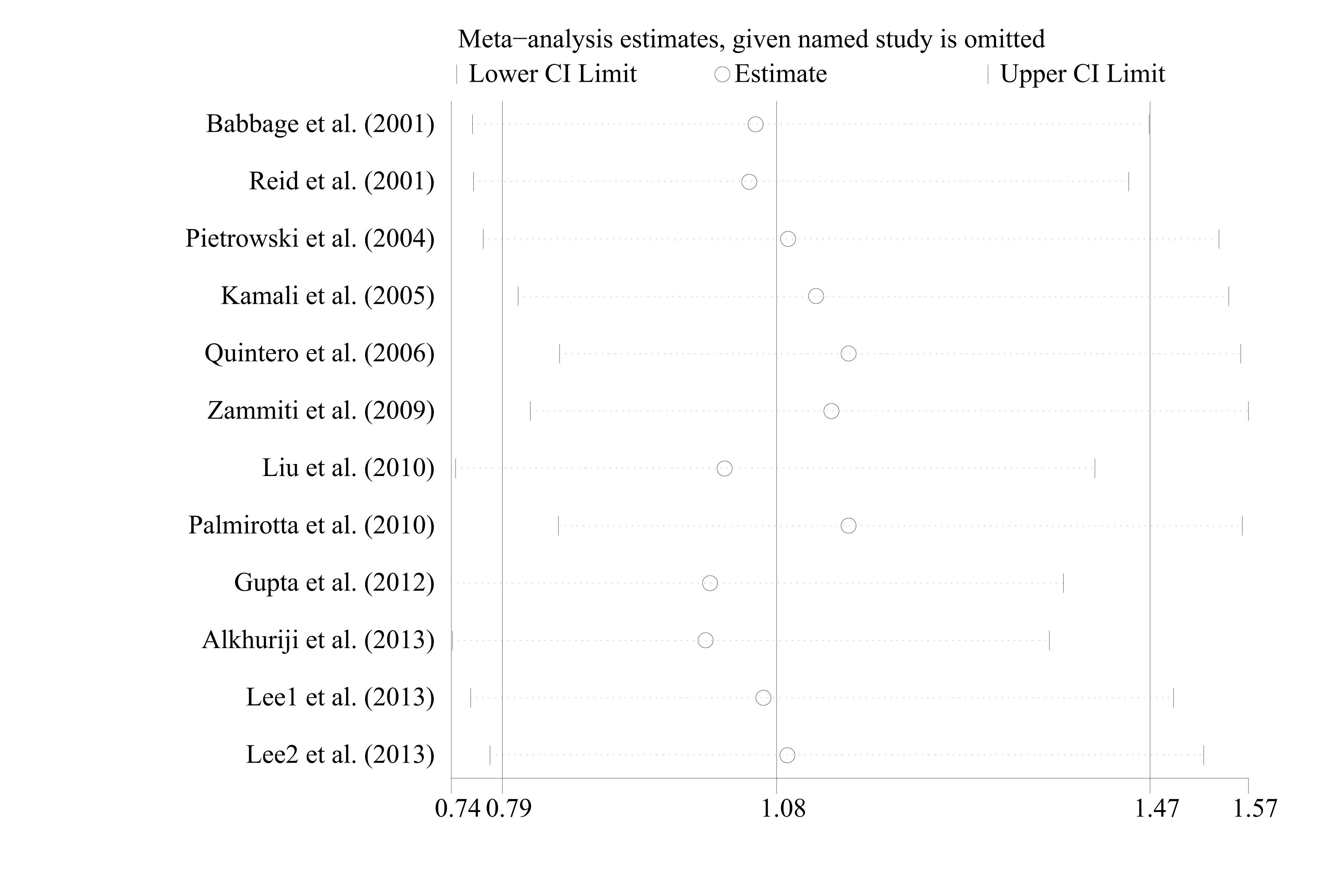

Supplement: S2 Fig — (TIF) [file pone.0166892.s002.tif]

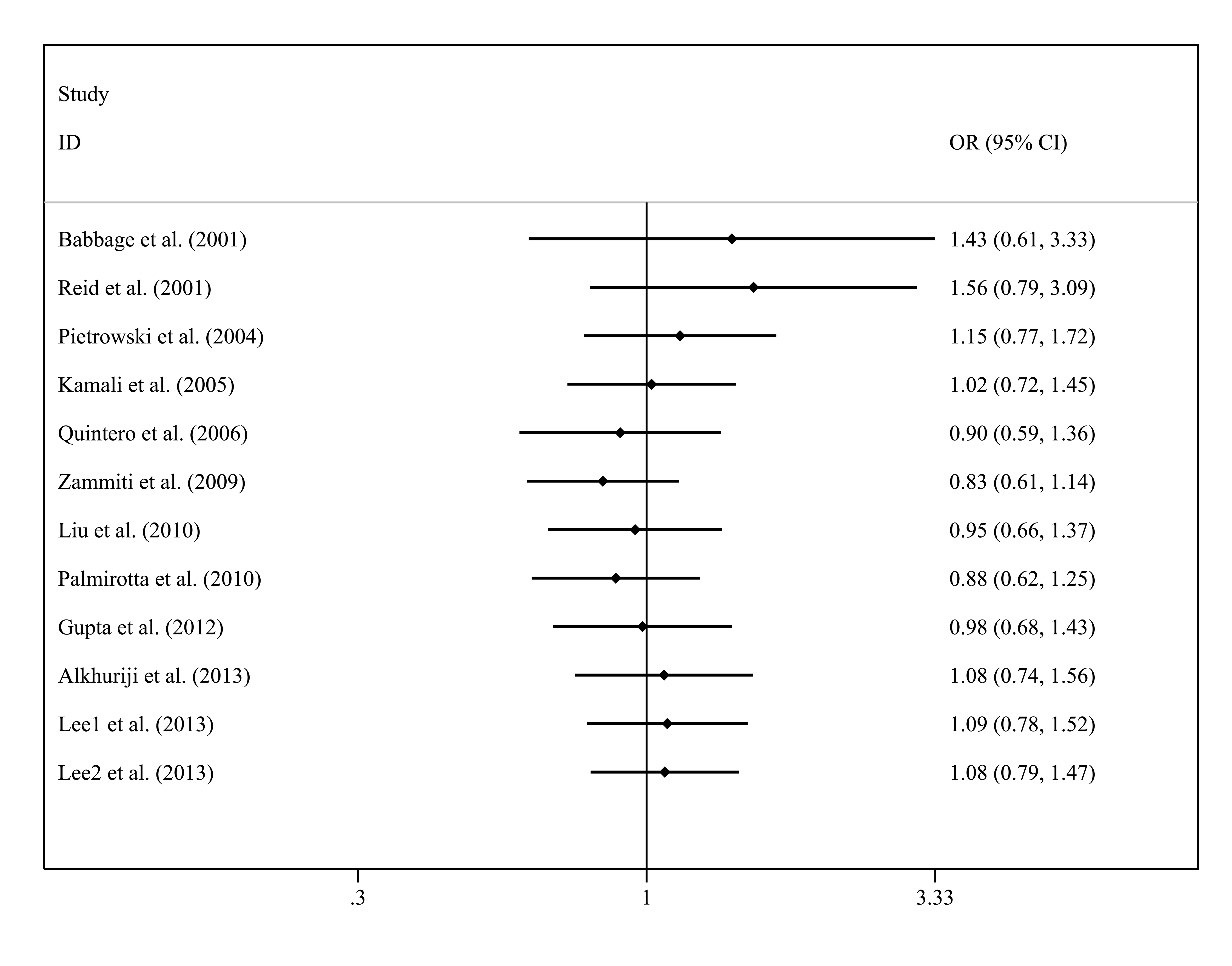

Supplement: S3 Fig — (TIF) [file pone.0166892.s003.tif]

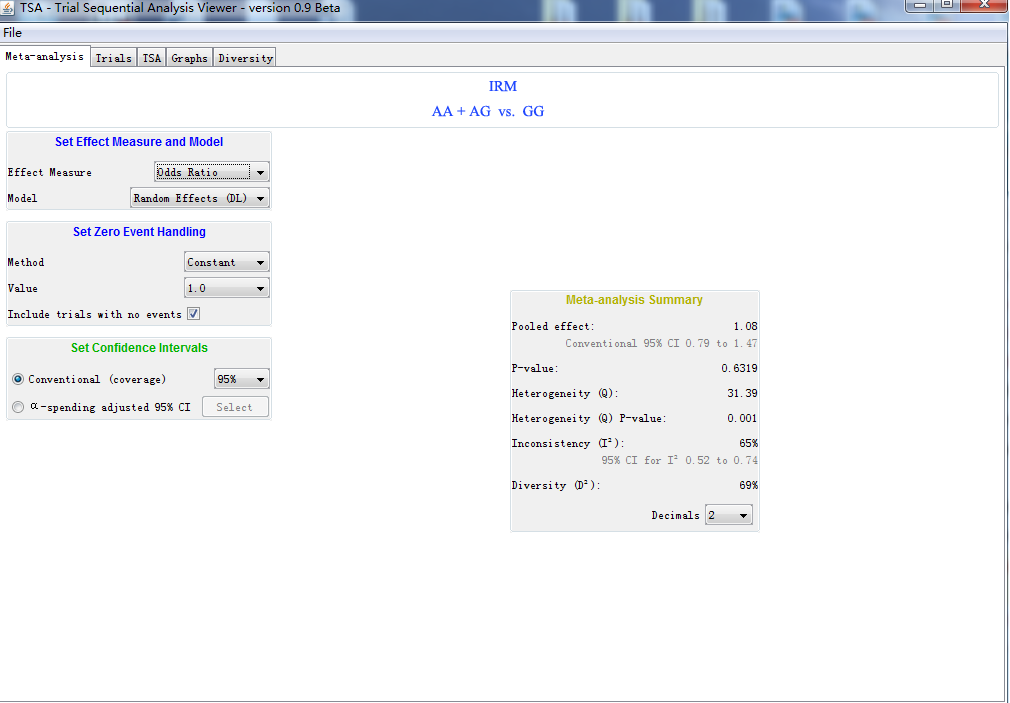

Supplement: S4 Fig — (TIF) [file pone.0166892.s004.tif]
