# Supplementary material for: No Association between TNF-α -308G/A Polymorphism and Idiopathic Recurrent Miscarriage: A Systematic Review with Meta-Analysis and Trial Sequential Analysis
Source: PLoS One. 2016 Nov 28;11(11):e0166892. doi: 10.1371/journal.pone.0166892 (PMC5125640; doi:10.1371/journal.pone.0166892)
Supplement: S1 File — (PDF) [file pone.0166892.s005.pdf]

Search Strategy 1

Electronic databases	PubMed, Embase, and the Cochrane Library
Search terms #1	tumor necrosis factor[Title/Abstract] OR TNF[Title/Abstract] OR Cachectin[Title/Abstract]
Search terms #2	polymorphism[Title/Abstract] OR SNP[Title/Abstract] OR genotype[Title/Abstract] OR mutation[Title/Abstract] OR variation[Title/Abstract] OR variant[Title/Abstract]
Search terms #3	abortion[Title/Abstract] OR miscarriage[Title/Abstract] OR pregnancy loss[Title/Abstract] OR fetal loss[Title/Abstract] OR fetal death[Title/Abstract] OR fetal lethality[Title/Abstract] OR fetus wastage[Title/Abstract] OR fetus death[Title/Abstract]
Search terms #4	Search terms #1 AND search terms #2 AND search terms #3

Search Strategy 2

Electronic databases	PubMed, Embase, and the Cochrane Library
Search terms #1	rs1800629
Search terms #2	abortion[Title/Abstract] OR miscarriage[Title/Abstract] OR pregnancy loss[Title/Abstract] OR fetal loss[Title/Abstract] OR fetal death[Title/Abstract] OR fetal lethality[Title/Abstract] OR fetus wastage[Title/Abstract] OR fetus death[Title/Abstract]
Search terms #3	Search terms #1 AND search terms #2

No new literature was found in Search Strategy 2.

PubMed Home | More Resources | Help

PubMed Advanced Search Builder

Tutorial

Query #1 deleted.

Use the builder below to create your search

Edit

Clear

Builder

All Fields ▼ Show index list

AND ▼ All Fields ▼ Show index list

Search or Add to history

History

Download history Clear history

Search	Add to builder	Query	Items found
#7	Add	Search **(((((((((abortion[Title/Abstract]) OR miscarriage[Title/Abstract]) OR pregnancy loss[Title/Abstract]) OR fetal loss[Title/Abstract]) OR fetal death[Title/Abstract]) OR fetal lethality[Title/Abstract]) OR fetus wastage[Title/Abstract]) OR fetus death[Title/Abstract])) AND ((((((polymorphism[Title/Abstract]) OR SNP[Title/Abstract]) OR genotype[Title/Abstract]) OR mutation[Title/Abstract]) OR variation[Title/Abstract]) OR variant[Title/Abstract])) AND (((tumor necrosis factor[Title/Abstract]) OR TNF[Title/Abstract]) OR Cachectin[Title/Abstract])**	35
#5	Add	Search **(((((((abortion[Title/Abstract]) OR miscarriage[Title/Abstract]) OR pregnancy loss[Title/Abstract]) OR fetal loss[Title/Abstract]) OR fetal death[Title/Abstract]) OR fetal lethality[Title/Abstract]) OR fetus wastage[Title/Abstract]) OR fetus death[Title/Abstract]**	64979
#6	Add	Search **(((((polymorphism[Title/Abstract]) OR SNP[Title/Abstract]) OR genotype[Title/Abstract]) OR mutation[Title/Abstract]) OR variation[Title/Abstract]) OR variant[Title/Abstract]**	947000
#2	Add	Search **((tumor necrosis factor[Title/Abstract]) OR TNF[Title/Abstract]) OR Cachectin[Title/Abstract]**	166268

Embase®

#22	#5 AND #12 AND #21	125
#21	#13 OR #14 OR #15 OR #16 OR #17 OR #18 OR #19 OR #20	128729
#20	'fetus death'	22880
#19	'fetal lethality'	70
#18	'fetal death'	7286
#17	'fetus wastage'	4782
#16	'fetal loss'	4181
#15	'pregnancy loss'	6837
#14	miscarriage	12599
#13	abortion	100689
#12	#6 OR #7 OR #8 OR #9 OR #10 OR #11	1714047
#11	variant	185088
#10	variation	506695
#9	mutation	721740
#8	genotype	344722
#7	snp	63557
#6	polymorphism	348381
#5	#2 OR #3 OR #4	309850
#4	'cachectin'	472
#3	'tnf'	172181
#2	'tumor necrosis factor'	269808

Elsevier logo

© 2016 RELX Intellectual Properties SA. All rights reserved.
Embase, RELX Group and the RE symbol are trade marks of RELX Intellectual Properties SA, used under license.

Cochrane Library

Trusted evidence.
Informed decisions.
Better health.

Log in / Register

Search | Search Manager | Medical Terms (MeSH) | Browse

Search All Text ▼ abortion or miscarriage or pregnancy loss or fetal loss or fetal death or fetal lethality o

Go Save

AND ▼ Search All Text ▼ polymorphism OR SNP OR genotype OR mutation OR variation OR variant

Add to Search Manager

AND ▼ Search All Text ▼ tumor necrosis factor OR TNF OR Cachectin

Search Limits Search Help (Word variations have been searched)

Clear

All Results (131)

- Cochrane Reviews (126)
  - All
  - Review
  - Protocol
- Other Reviews (0)
- Trials (2)
- Methods Studies (0)
- Technology Assessments (0)
- Economic Evaluations (0)
- Cochrane Groups (3)

- All
- Current Issue

Cochrane Central Register of Controlled Trials : Issue 6 of 12, June 2016

There are **2** results from **951138** records for your search on '**abortion or miscarriage or pregnancy loss or fetal loss or fetal death or fetal lethality or fetus wastage or fetus death and polymorphism OR SNP OR genotype OR mutation OR variation OR variant and tumor necrosis factor OR TNF OR Cachectin** in **Trials**'

Sort by Relevance: high to low ▼

Select all | Export all | Export selected

A **tumor necrosis factor** -alpha promoter **polymorphism** and pregnancy complications: results of a prospective cohort study in 1652 pregnant women.
Stonek F , Bentz EK , Hafner E , Metzenbauer M , Philipp K , Hefler LA and Tempfer CB
Reproductive sciences (Thousand Oaks, Calif.), 2007, 14(5), 425
Publication Year: 2007

Plasma **TNF** -α levels are higher in early pregnancy in patients with secondary compared with primary recurrent **miscarriage** .
Piosik ZM , Goegebeur Y , Klitkou L , Steffensen R and Christiansen OB

PubMed Advanced Search Builder

Tutorial

Use the builder below to create your search

Edit

Clear

Builder

All Fields ▼ Show index list

AND ▼ All Fields ▼ Show index list

Search or Add to history

History

Download history Clear history

Search	Add to builder	Query	Items found
#3	Add	Search **((((((((abortion[Title/Abstract]) OR miscarriage[Title/Abstract]) OR pregnancy loss[Title/Abstract]) OR fetal loss[Title/Abstract]) OR fetal death[Title/Abstract]) OR fetal lethality[Title/Abstract]) OR fetus wastage[Title/Abstract]) OR fetus death[Title/Abstract])) AND rs1800629**	2
#2	Add	Search **(((((((abortion[Title/Abstract]) OR miscarriage[Title/Abstract]) OR pregnancy loss[Title/Abstract]) OR fetal loss[Title/Abstract]) OR fetal death[Title/Abstract]) OR fetal lethality[Title/Abstract]) OR fetus wastage[Title/Abstract]) OR fetus death[Title/Abstract]**	65063
#1	Add	Search **rs1800629**	261

Don't miss the next webinar! Learn how drug analytics using Embase can inform drug development, repositioning and safety on July 27 at 4:00 pm CET

#9 AND #10

Results Filters

+ Expand — Collapse all

Apply >

- Sources
- Drugs
- Diseases
- Devices
- Floating Subheadings
- Age
- Gender
- Study types
- Publication types
- Journal titles
- Publication years
- Authors
- Conference Abstracts
- Drug Trade Names
- Drug Manufacturers
- Device Trade Names
- Device Manufacturers

Apply >

#11	#9 AND #10
#10	'rs1800629'
#9	#1 OR #2 OR #3 OR #4 OR #5 OR #6 OR #
#8	'fetus death'
#7	'fetus wastage'
#6	'fetal lethality'
#5	'fetal death'
#4	'fetal loss'
#3	'miscarriage'
#2	'pregnancy loss'
#1	'abortion'

2 results for search #11 |

Show all abstracts 1 — 2

Select number of items ▼ Selected: 0 (clear) S

1 Association of TNF-αα genetic polymorphisms with recurrent pregnancy loss risk: A systematic review and meta-analysis

Li H.-H., Xu X.-H., Tong J., Zhang K.-Y., Zhang C., Chen Z.-J.

Reproductive Biology and Endocrinology 2016 **14:1** Article Number 6

Embase

Abstract Index Terms

> View Full Text

2 Tumor necrosis factor-308 polymorphism increases the embryo implantation rate in women undergoing in vitro fertilization

Vialard F., El Sirkasi M., Tronchon V., Boudjenah R., Molina-Gomes D., Bergere M., Mauduit C., Wainer R., Selva J., Benahmed M.

Human Reproduction 2013 **28:10** (2774-2783)

Embase MEDLINE Cited by: 4

Abstract Index Terms

> View Full Text

Records per page 25 ▼

Wiley Online Library

Cochrane Library

Trusted evidence.
Informed decisions.
Better health.

Log in / Register

Search | Search Manager | Medical Terms (MeSH) | Browse

Search All Text ▼ | abortion or miscarriage or pregnancy loss or fetal loss or fetal death or fetal lethality o | Go | Save

AND ▼ | Search All Text ▼ | rs1800629 | Add to Search Manager

Search Limits | Search Help | (Word variations have been searched)

Clear

All Results (0)

- Cochrane Reviews (0)
  - All
  - Review
  - Protocol
- Other Reviews (0)
- Trials (0)
- Methods Studies (0)
- Technology Assessments (0)
- Economic Evaluations (0)
- Cochrane Groups (0)

Cochrane Database of Systematic Reviews : Issue 7 of 12, July 2016

Issue ***updated daily*** throughout month

There are **0** results from **0** records for your search on '**abortion or miscarriage or pregnancy loss or fetal loss or fetal death or fetal lethality or fetus wastage or fetus death and rs1800629** '
