# Supplementary material for: No Association between TNF-α -308G/A Polymorphism and Idiopathic Recurrent Miscarriage: A Systematic Review with Meta-Analysis and Trial Sequential Analysis
Source: PLoS One. 2016 Nov 28;11(11):e0166892. doi: 10.1371/journal.pone.0166892 (PMC5125640; doi:10.1371/journal.pone.0166892)
Supplement: S2 File — (PDF) [file pone.0166892.s006.pdf]

Data obtained via e-mail

There were three relevant articles lack of sufficient data [31, 33, 34], however, after reaching out to the authors via e-mail, only one replied [34].

The following table details the original data in manuscript by Kamali et al. (Kamali-Sarvestani E, Zolghadri J, Gharesi-Fard B, Sarvari J. Cytokine gene polymorphisms and susceptibility to recurrent pregnancy loss in Iranian women. J Reprod Immunol. 2005;65(2):171-8. Epub 2005/04/07. doi: 10.1016/j.jri.2005.01.008. PubMed PMID: 15811521.).

Table 3
Distribution of cytokine genotypes in control subjects and in patients with RPL

Genotype (phenotype)	Number of patients (frequency)	Number of controls (frequency)	p-Value
TNF-α −308			
AA/AG (high)	14 (0.11)	21 (0.15)	0.42
GG (low)	117 (0.89)	122 (0.85)	

Dr. Eskandar Kamali-Sarvestani replied soon after our e-mail.

"We did not detect TNF-alpha AA genotype in our patients and controls. In fact, in patients and controls mentioned in the table with AA+AG genotypes, we only had AG genotype. I hope this detail will be helpful."

Then in this manuscript, the genotype frequencies were obtained.

Excluded Studies before data extraction: categorized by reasons

Studies without clear data

1. Baxter N, Sumiya M, Cheng S, Erlich H, Regan L, Simons A, et al. Recurrent miscarriage and variant alleles of mannose binding lectin, tumour necrosis factor and lymphotoxin alpha genes. Clin Exp Immunol. 2001;126(3):529-34. Epub 2001/12/12. PubMed PMID: 11737072; PubMed Central PMCID: PMCPmc1906238.
2. Daher S, Shulzhenko N, Morgun A, Mattar R, Rampim GF, Camano L, et al. Associations between cytokine gene polymorphisms and recurrent pregnancy loss. J Reprod Immunol. 2003;58(1):69-77. Epub 2003/03/01. PubMed PMID: 12609526.
3. Prigoshin N, Tambutti M, Larriba J, Gogorza S, Testa R. Cytokine gene polymorphisms in recurrent pregnancy loss of unknown cause. Am J Reprod Immunol. 2004;52(1):36-41. Epub 2004/06/25. doi: 10.1111/j.1600-0897.2004.00179.x. PubMed PMID: 15214940.

Study populations overlapped

4. Zammiti W, Mtiraoui N, Khairi H, Gris JC, Almawi WY, Mahjoub T. Associations between tumor necrosis factor-alpha and lymphotoxin-alpha polymorphisms and idiopathic recurrent miscarriage. Reproduction. 2008;135(3):397-403. Epub 2008/02/27. doi: 10.1530/rep-07-0322. PubMed PMID: 18299433.

Study focusing on TNF-α -308G/A polymorphism and antiphospholipid syndrome associated miascarriages

5. Bertolaccini ML, Atsumi T, Lanchbury JS, Caliz AR, Katsumata K, Vaughan RW, et al. Plasma tumor necrosis factor alpha levels and the -238*A promoter polymorphism in patients with antiphospholipid syndrome. Thromb Haemost. 2001;85(2):198-203. Epub 2001/03/15. PubMed PMID: 11246532.

Study focusing on TNF-α -308G/A polymorphism and miascarriages associated with HEV infection during pregnancy

6. Devi SG, Kumar A, Kar P, Husain SA, Sharma S. Association of pregnancy outcome with cytokine gene polymorphisms in HEV infection during pregnancy. J Med Virol. 2014;86(8):1366-76. Epub 2014/03/13. doi: 10.1002/jmv.23925. PubMed PMID: 24610501.

Study focusing on TNF-α -308G/A polymorphism and pregnancy complications such as preeclampsia and preterm delivery

7. Stonek F, Bentz EK, Hafner E, Metzenbauer M, Philipp K, Hefler LA, et al. A tumor necrosis factor-alpha promoter polymorphism and pregnancy complications: results of a prospective cohort study in 1652 pregnant women. Reprod Sci. 2007;14(5):425-9. Epub 2007/10/05. doi: 10.1177/1933719107305213. PubMed PMID: 17913961.

Study focusing on TNF-α -308G/A polymorphism and embryo implantation

8. Vialard F, El Sirkasi M, Tronchon V, Boudjenah R, Molina-Gomes D, Bergere M, et al. Tumor necrosis factor-308 polymorphism increases the embryo implantation rate in women undergoing in vitro fertilization. Hum Reprod. 2013;28(10):2774-83. Epub 2013/08/03. doi: 10.1093/humrep/det264. PubMed PMID: 23906902.

other irrelevant articles

9. Jang HG, Choi Y, Kim JO, Jeon YJ, Rah H, Cho SH, et al. Polymorphisms in tumor necrosis factor-alpha (-863C>A, -857C>T and +488G>A) are associated with idiopathic recurrent pregnancy loss in Korean women. Hum Immunol. 2016;77(6):506-11. Epub 2016/04/17. doi: 10.1016/j.humimm.2016.04.012. PubMed PMID: 27083536.

10. Parveen F, Agrawal S. A study of forty-seven single nucleotide polymorphisms among recurrent miscarriage using classification and regression tree analysis. Am J Reprod Immunol. 2013;70(6):529-37. Epub 2013/08/02. doi: 10.1111/aji.12152. PubMed PMID: 23902348.

11. Piosik ZM, Goegebeur Y, Klitkou L, Steffensen R, Christiansen OB. Plasma TNF-alpha levels are higher in early pregnancy in patients with secondary compared with primary recurrent miscarriage. Am J Reprod Immunol. 2013;70(5):347-58. Epub 2013/05/10. doi: 10.1111/aji.12135. PubMed PMID: 23656493.
