# Supplementary material for: No Association between TNF-α -308G/A Polymorphism and Idiopathic Recurrent Miscarriage: A Systematic Review with Meta-Analysis and Trial Sequential Analysis
Source: PLoS One. 2016 Nov 28;11(11):e0166892. doi: 10.1371/journal.pone.0166892 (PMC5125640; doi:10.1371/journal.pone.0166892)
Supplement: S3 File — (PDF) [file pone.0166892.s007.pdf]

Tests for Publication Bias

Egger's test

Std_Eff	Coef.	Std. Err.	t	P>|t|	[95% Conf.	Interval]
slope	.2541678	.5065475	0.50	0.627	-.8744903	1.382826
bias	-.5294831	1.660906	-0.32	0.756	-4.230213	3.171247

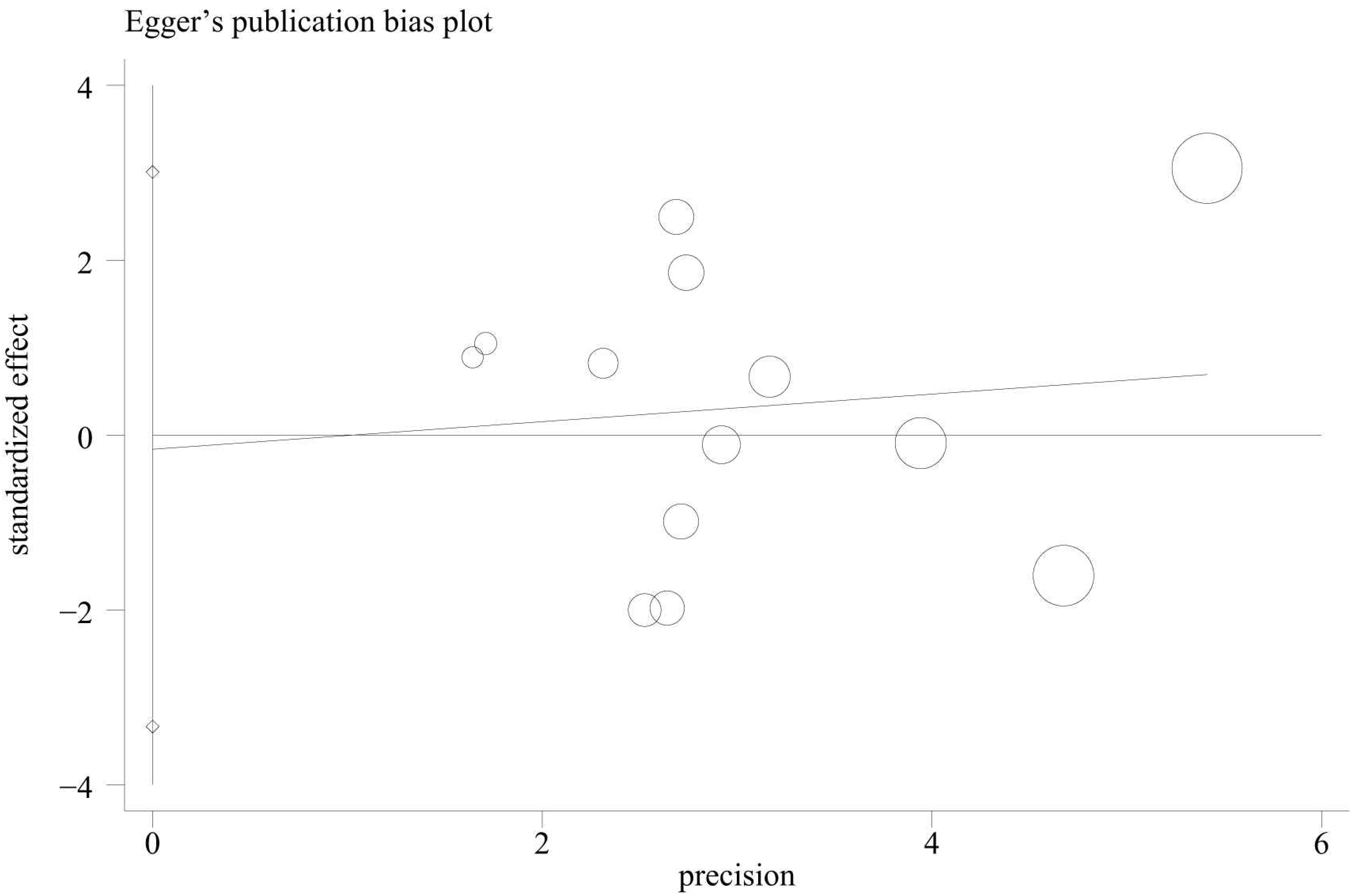
Egger's publication bias plot
standardized effect
precision
4
2
0
−2
−4
0
2
4
6
