# Supplementary material for: No Association between TNF-α -308G/A Polymorphism and Idiopathic Recurrent Miscarriage: A Systematic Review with Meta-Analysis and Trial Sequential Analysis
Source: PLoS One. 2016 Nov 28;11(11):e0166892. doi: 10.1371/journal.pone.0166892 (PMC5125640; doi:10.1371/journal.pone.0166892)
Supplement: S4 File — (PDF) [file pone.0166892.s008.pdf]

Additional meta-analysis with dubious data provided in a previous meta-analysis

After looking into previous meta-analyses with great care, we found there were some data that cannot be found in the original manuscripts, but were listed out and analyzed in meta-analysis of 2012 (Zhang B, Liu T, Wang Z. Association of tumor necrosis factor-α gene promoter polymorphisms (-308G/A,-238G/A) with recurrent spontaneous abortion: a meta-analysis. Human immunology. 2012;73(5):574-9.) (**Table 4,** [b]).

For the data by Baxter et al. extracted in two meta-analyses [47, 48], genotype frequencies were different, and all of them cannot be found in the original manuscript (Table 4). Therefore, we did not include them in this additional meta-analysis.

For others, frequencies of AA+GA and GG were provided in the original manuscript. And the possibility exists that the original data of AA or GA genotype frequencies may be obtained from authors directly. Therefore we adopt them in this additional meta-analysis.

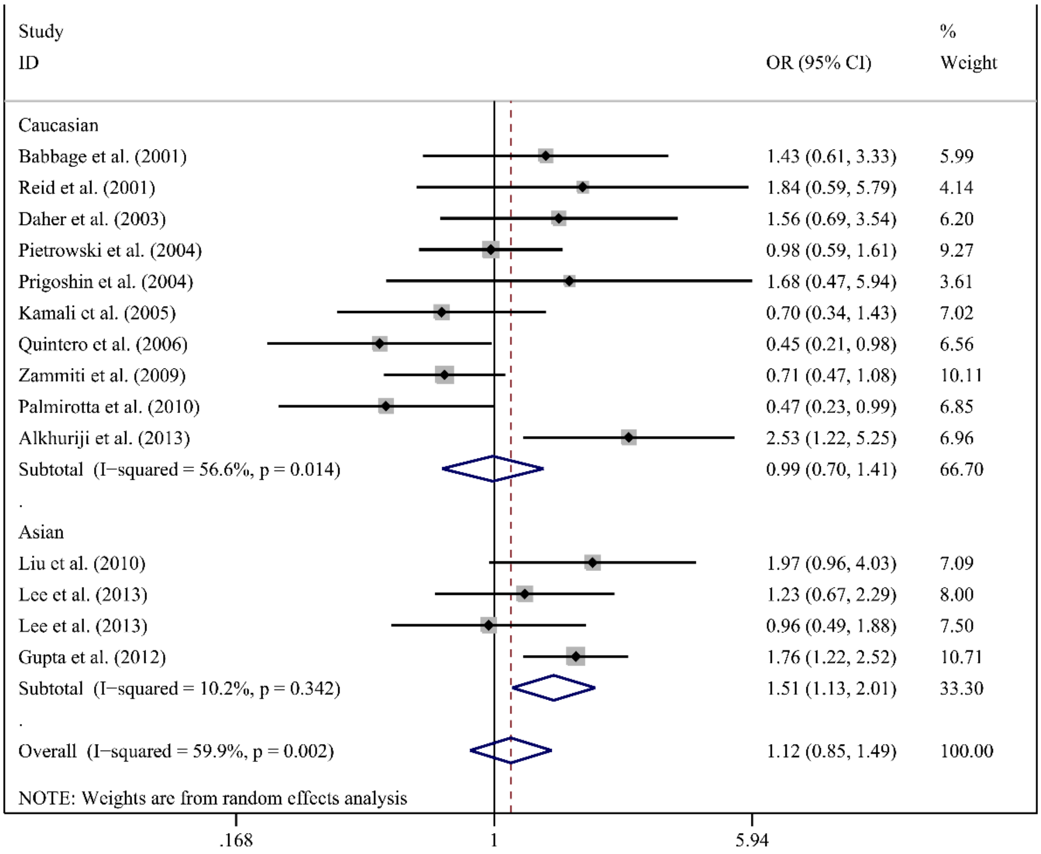

Fig. 1 Forest plot for the association between -308A/G polymorphism and IRM risk in dominant model.

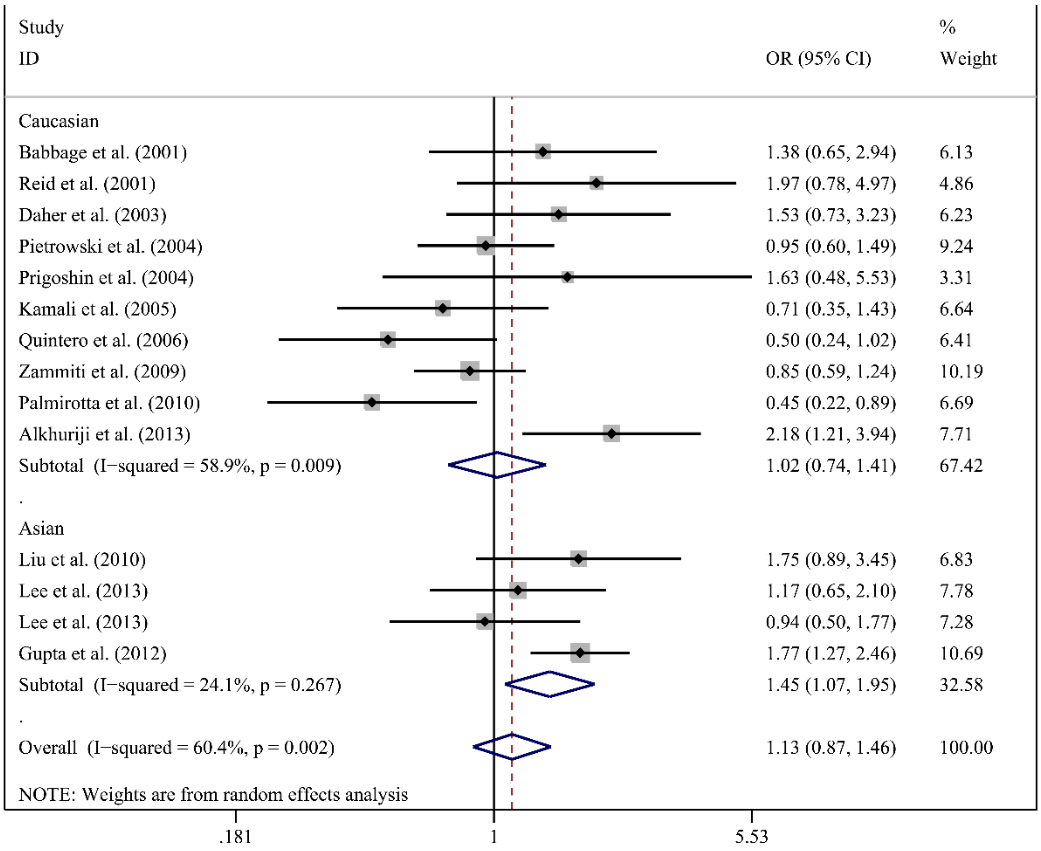

Fig. 2 Forest plot for the association between -308A/G polymorphism and IRM risk classified by ethnicity in alleles model.

No significant association was found.
