# Supplementary material for: No Association between TNF-α -308G/A Polymorphism and Idiopathic Recurrent Miscarriage: A Systematic Review with Meta-Analysis and Trial Sequential Analysis
Source: PLoS One. 2016 Nov 28;11(11):e0166892. doi: 10.1371/journal.pone.0166892 (PMC5125640; doi:10.1371/journal.pone.0166892)
Supplement: S5 File — (PDF) [file pone.0166892.s009.pdf]

Table X1

Primary characteristics of the 13 studies included in the meta-analysis (with the Chinese article)

Study	Study location	Ethnicity	Source of control	Age		Definition of IRM	Samples size			Genotyping method	Case		Control		Quality score
				Case	Control		Case	Control	Total		Genotype frequency AA/AG/GG	HWE *P*	Genotype frequency AA/AG/GG	HWE *P*	
Babbage,2001 [28]	UK	Caucasian	HB	21-45	30-58	≥3	43	73	116	PCR-ASA	1/12/30	0.876	3/14/56	0.107	7
Reid,2001 [29]	UK	Caucasian	HB	-	-	≥2	17	43	60	PCR-RFLP	2/6/9	0.536	1/13/29	0.744	2
Pietrowski,2004 [30]	Germany	Caucasian	PB	33	58	≥3	168	212	380	PCR	2/33/133	0.977	4/41/167	0.432	7
Kamali,2005 [31]	Iran	Caucasian	PB	18-42	-	≥3	131	143	274	PCR-ASO	0/14/117	0.518	0/21/122	0.343	6
Quintero,2006[a] [32]	Mexico	Caucasian	PB	-	-	≥3	122	214	336	PCR	1/8/113	0.067	2/30/182	0.544	5
Zhou,2006[b] [X]	China	Asian	HB	27	-	≥3	50	50	100	PCR-RFLP	0/8/42	0.539	0/5/45	0.710	7
Zammiti,2009 [33]	Tunisia	Caucasian	PB	29	29	≥3	372	274	646	PCR-RFLP	14/39/319	0	5/47/222	0.187	10
Liu,2010 [34]	China	Asian	HB	30	29	≥2	132	152	284	PCR	0/22/110	0.296	1/13/138	0.276	6
Palmirotta,2010 [35]	Italy	Caucasian	PB	37	38	≥2	100	100	200	PCR	0/13/87	0.487	3/21/76	0.313	5
Gupta,2012 [36]	India	Asian	PB	-	-	≥3	300	500	800	PCR-RFLP	9/62/229	0.067	5/70/425	0.274	7
Alkhuriji,2013 [37]	Saudi Arabia	Caucasian	HB	34	-	≥3	65	65	130	PCR-SSP	8/24/33	0.282	4/14/47	0.059	7
Lee1,2013 [38]	South Korea	Asian	PB	33	33	≥2	187	236	423	PCR-RFLP	1/21/165	0.711	2/21/213	0.083	8
Lee2,2013 [38]	South Korea	Asian	PB	33	33	≥3	170	236	406	PCR-RFLP	1/15/154	0.353	2/21/213	0.083	9

PCR-RFLP, polymerase chain reaction-restriction fragment length polymorphism; PCR-ASA, polymerase chain reaction-allele specific amplification; PCR-ASO, polymerase chain reaction-allele specific oligonucleotide; PCR-SSP, polymerase chain reaction-sequence specific primers; HWE, Hardy-Weinberg equilibrium; PB, population based; HB, hospital based; [a], this article is in Spanish with English abstract; [b], this article is in Chinese without English abstract.

X. Zhou Y, Li JY, Song LY, Zhou M, Chen SQ. Study on plasma TNF level and TNF alpha -308 polymorphisms in patients with unexplained repeated spontaneous abortion. J Mod Clin Med Bioeng 2006;12:32–4.

Table X2

Primary results of overall meta-analyses and subgroup analyses with the Chinese article.

Comparison	Group	No. of studies	Test of association			Effect model	Test of heterogeneity	
			OR	95%CI	P_{OR}		I^2 (%)	P_Q
**-308G/A (rs1800629)**		**13**						
**AA vs.GG**	**Overall**	**11[b]**	**1.08**	**(0.80,1.47)**	**0.608**	**random**	**61.1**	**0.002**
**AG vs.GG**	**Overall**	**13**	**1.49**	**(0.93,2.40)**	**0.098**	**fixed**	**4.7**	**0.399**
**AA vs.AG + GG**	**Overall**	**11[b]**	**1.27**	**(0.77,2.10)**	**0.344**	**fixed**	**0**	**0.692**
**AA + AG vs.GG**	**Overall**	**13**	**1.10**	**(0.82,1.48)**	**0.531**	**random**	**62.4**	**0.001**
Ethnicity	Asian	5	1.53	**(1.19,1.98)[a]**	0.001	fixed	0	0.497
	Caucasian	8	0.91	(0.62,1.34)	0.639	random	61.1	0.012
Definition of IRM	≥3 miscarriages	9	1.08	(0.76,1.54)	0.683	random	66	0.003
	≥2 miscarriages	4	1.17	(0.61,2.24)	0.637	random	64.2	0.039
Source of control	HB	5	1.94	**(1.32,2.85)[a]**	0.001	fixed	0	0.899
	PB	8	0.87	(0.61,1.23)	0.436	random	67	0.003
Total sample size	＜150	4	1.92	**(1.22,3.03)[a]**	0.005	fixed	0	0.786
	＞150	9	0.94	(0.67,1.32)	0.725	random	67.5	0.002
Score	≤6	5	0.85	(0.46,1.59)	0.612	random	67.4	0.016
	＞6	8	1.25	(0.91,1.72)	0.167	random	55.9	0.026
Galbraith plot	insiders[c]	9	1.05	(0.83,1.33)	0.699	fixed	28.8	0.189
	outliers[d]	4	1.11	(0.56,2.20)	0.760	random	85.3	0
**A vs. G**	**Overall**	**13**	**1.10**	**(0.84,1.44)**	**0.500**	**random**	**62.7**	**0.001**
Ethnicity	Asian	5	1.50	**(1.18,1.90)[a]**	0.001	fixed	0	0.409
	Caucasian	8	0.94	(0.66,1.44)	0.728	random	63.4	0.008
Definition of IRM	≥3 miscarriages	9	1.09	(0.80,1.49)	0.588	random	63.9	0.005
	≥2 miscarriages	4	1.13	(0.60,2.15)	0.706	random	69.8	0.019
Source of control	HB	5	1.77	**(1.27,2.47)[a]**	0.001	fixed	0	0.828
	PB	8	0.90	(0.64,1.24)	0.485	random	68.8	0.002
Total sample size	＜150	4	1.78	**(1.21,2.61)[a]**	0.004	fixed	0	0.684
	＞150	9	0.95	(0.69,1.30)	0.743	random	67.7	0.002
Score	≤6	5	1.24	(0.95,1.63)	0.631	random	69.9	0.010
	＞6	8	0.86	(0.47,1.57)	0.116	random	51	0.046
Galbraith plot	insiders[c]	9	1.03	(0.82,1.28)	0.816	fixed	23.9	0.231
	outliers[d]	4	1.13	(0.62,2.07)	0.688	random	85.2	0

CI, confidence interval; OR, odds ratio; IRM, idiopathic recurrent miscarriage; [a], significant results; [b], two studies [34, X] are not analyzed due to 0 AA genotype in both case and control group; Of the 13 studies included in this meta-analysis, 9 studies from 8 articles [28-32, 34, 38, X] are insiders[c], 4 studies [33, 35-37] are outliners[d] in Galbraith plot.

X. Zhou Y, Li JY, Song LY, Zhou M, Chen SQ. Study on plasma TNF level and TNF alpha -308 polymorphisms in patients with unexplained repeated spontaneous abortion. J Mod Clin Med Bioeng 2006;12:32–4.

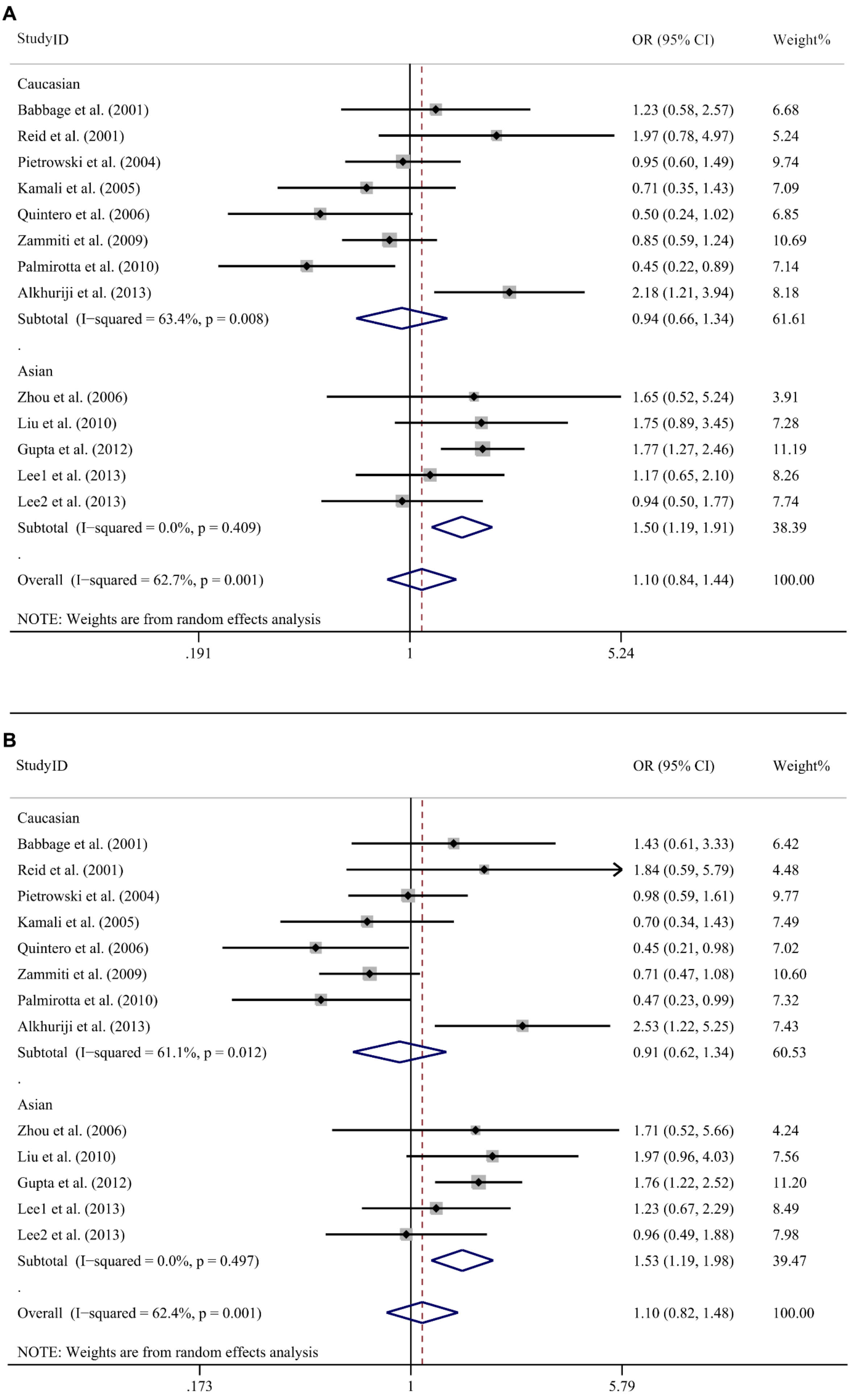
A
StudyID
OR (95% CI)
Weight%
Caucasian
Babbage et al. (2001) 1.23 (0.58, 2.57) 6.68
Reid et al. (2001) 1.97 (0.78, 4.97) 5.24
Pietrowski et al. (2004) 0.95 (0.60, 1.49) 9.74
Kamali et al. (2005) 0.71 (0.35, 1.43) 7.09
Quintero et al. (2006) 0.50 (0.24, 1.02) 6.85
Zammiti et al. (2009) 0.85 (0.59, 1.24) 10.69
Palmirotta et al. (2010) 0.45 (0.22, 0.89) 7.14
Alkhuriji et al. (2013) 2.18 (1.21, 3.94) 8.18
Subtotal (I−squared = 63.4%, p = 0.008) 0.94 (0.66, 1.34) 61.61
.
Asian
Zhou et al. (2006) 1.65 (0.52, 5.24) 3.91
Liu et al. (2010) 1.75 (0.89, 3.45) 7.28
Gupta et al. (2012) 1.77 (1.27, 2.46) 11.19
Lee1 et al. (2013) 1.17 (0.65, 2.10) 8.26
Lee2 et al. (2013) 0.94 (0.50, 1.77) 7.74
Subtotal (I−squared = 0.0%, p = 0.409) 1.50 (1.19, 1.91) 38.39
.
Overall (I−squared = 62.7%, p = 0.001) 1.10 (0.84, 1.44) 100.00
NOTE: Weights are from random effects analysis
.191
1
5.24
B
StudyID
OR (95% CI)
Weight%
Caucasian
Babbage et al. (2001) 1.43 (0.61, 3.33) 6.42
Reid et al. (2001) 1.84 (0.59, 5.79) 4.48
Pietrowski et al. (2004) 0.98 (0.59, 1.61) 9.77
Kamali et al. (2005) 0.70 (0.34, 1.43) 7.49
Quintero et al. (2006) 0.45 (0.21, 0.98) 7.02
Zammiti et al. (2009) 0.71 (0.47, 1.08) 10.60
Palmirotta et al. (2010) 0.47 (0.23, 0.99) 7.32
Alkhuriji et al. (2013) 2.53 (1.22, 5.25) 7.43
Subtotal (I−squared = 61.1%, p = 0.012) 0.91 (0.62, 1.34) 60.53
.
Asian
Zhou et al. (2006) 1.71 (0.52, 5.66) 4.24
Liu et al. (2010) 1.97 (0.96, 4.03) 7.56
Gupta et al. (2012) 1.76 (1.22, 2.52) 11.20
Lee1 et al. (2013) 1.23 (0.67, 2.29) 8.49
Lee2 et al. (2013) 0.96 (0.49, 1.88) 7.98
Subtotal (I−squared = 0.0%, p = 0.497) 1.53 (1.19, 1.98) 39.47
.
Overall (I−squared = 62.4%, p = 0.001) 1.10 (0.82, 1.48) 100.00
NOTE: Weights are from random effects analysis
.173
1
5.79
