# Supplementary material for: No Association between TNF-α -308G/A Polymorphism and Idiopathic Recurrent Miscarriage: A Systematic Review with Meta-Analysis and Trial Sequential Analysis
Source: PLoS One. 2016 Nov 28;11(11):e0166892. doi: 10.1371/journal.pone.0166892 (PMC5125640; doi:10.1371/journal.pone.0166892)
Supplement: S2 Table — (DOCX) [file pone.0166892.s011.docx]

**Meta-analysis on Genetic Association Studies Checklist | PLOS ONE**

|  | Item | Section name and paragraph number within manuscript |
| --- | --- | --- |
|  | **Introduction** |  |
| 1 | Provide a detailed justification for the polymorphism studied; if a single polymorphism was analyzed, give details as to why others were not included in the meta-analysis. | Introduction  Paragraph 3 Page 3 |
| 2 | Provide a detailed justification for the population(s) and clinical condition studied. | Introduction  Paragraph 1, 2 Page 3 |
|  | **Methods** |  |
| 3 | Provide full details of the search strategy employed; outline the full electronic search strategy –specific combination of keywords and any limits applied- for at least one database. Specify whether synonyms of polymorphisms/genes (e.g. SNP number) were searched. | Identification of eligible trials  Paragraph 4 Page 4  File S1 |
| 4 | Report full details on the inclusion and exclusion criteria applied for selecting studies.  *Please list the excluded articles and the reasons for exclusion of each article in a supplementary file.* | Inclusion and exclusion criteria  Paragraph 4 Page 4 |
| 5 | Provide details on how the quality of the studies included in the analyses was assessed. | Quality assessment  Paragraph 2 Page 5  Table 1 |
| 6 | Describe steps taken to contact study authors to identify additional studies and to request missing data. | Data extraction  Paragraph 1 Page 5  File S2 |
| 7 | Describe how environmental effects were adjusted for, if this adjustment was not conducted, outline the reasons for this. | N/A.  The influence of environmental factors on single nucleotide polymorphisms are complicated and immeasurable in different ethnic populations. |
| 8 | Describe the methods of handling heterogeneity/between-study variance. | Stastical analysis  Paragraph 3 Page 5 |
| 9 | Describe how the Hardy-Weinberg equilibrium and linkage disequilibrium were assessed. | Data extraction  Paragraph 5 Page 4-5 |
| 10 | Describe and justify the choice of model for the analyses (per-allele vs per-genotype vs genetic model-free, random effects vs fixed effects). | Stastical analysis  Paragraph 3 Page 5 |
| 11 | Describe whether a sensitivity analysis has been completed. | Stastical analysis  Paragraph 3 Page 5 |
| 12 | Describe whether an assessment of the effects of population stratification has been conducted. | Stastical analysis  Paragraph 3 Page 5 |
| 13 | Describe whether study-specific results have been assessed and if so the reasons for this (e.g. forest plot). | Stastical analysis  Paragraph 1 Page 6 |
|  | **Results** |  |
| 14 | Include flow diagram for the studies included in the meta-analysis as the first figure for the manuscript | Literature selection  Paragraph 2 Page 6  Fig 1 |
| 15 | Report details on allele/genotype prevalence. | Characteristics of included studies  Paragraph 3 Page 6  Table 2 |
| 16 | Report the effect size estimates and p values for each analysis. | Meta-analysis results  Paragraph 4 Page 6  Fig 2 Table 3 |
|  | **Discussion** |  |
| 17 | Discuss the limitations of the meta-analysis, including genotyping errors/bias and publication bias. | Discussion  Paragraph 2 Page 10 |
| 18 | If the meta-analysis identifies an association within a subgroup of the population studied but not another, discuss the implications of these results, and if applicable the possibility of subgroup-specific publication bias. | Discussion  Paragraph 2 Page 9 |
| 19 | Discuss the suitability of the sample size employed to the research question and the power of the study. | Discussion  Paragraph 2 Page 9 |
