# Supplementary material for: No Association between TNF-α -308G/A Polymorphism and Idiopathic Recurrent Miscarriage: A Systematic Review with Meta-Analysis and Trial Sequential Analysis
Source: PLoS One. 2016 Nov 28;11(11):e0166892. doi: 10.1371/journal.pone.0166892 (PMC5125640; doi:10.1371/journal.pone.0166892)
Supplement: S3 Table — (DOCX) [file pone.0166892.s012.docx]

Table S3 Inclusion criteria of all the studies included

| Study | location | Ethnicity | Inclusion criteria for Case |  | Inclusion criteria for Case |
| --- | --- | --- | --- | --- | --- |
| Babbage,2001 [28] | UK | Caucasian | Women (aged 21–45 years) who had experienced  at least three RPLs and in whom anatomical, hormonal, chromosomal, infectious or autoimmune causes including anti-phospholipid antibody syndrome had been excluded |  | Ward staff, aged 30–58 years, who had had at least two children (four had three children, one had four children and one had ﬁve children) and no known pregnancy losses. |
| Reid,2001 [29] | UK | Caucasian | Women attending the recurrent miscarriage clinic; Those with subsequently explained causes for RPL, such as a Robertsonian translocation, were excluded from further analysis. |  | Fertile women who had normal pregnancies and no complications. Women with a history of miscarriage or complications of pregnancy such as pre-eclampsia were excluded. |
| Pietrowski,2004 [30] | Germany | Caucasian | A diagnosis of idiopathic recurrent miscarriage was based on a documented history of at least three spontaneous, consecutive miscarriages before 20 weeks gestation with the same partner.  All underwent a standard diagnostic work-up to rule out a veriﬁable cause of recurrent miscarriage prior to inclusion into the study. |  | The control group consisted of 212 women with at least one live-birth and no history of miscarriage. To avoid confounding by genetic admixture, only women whose parents were of the same ethnicity were included in the study and control groups. |
| Kamali,2005 [31] | Iran | Caucasian | Women aged 18-42 years with at least three sequential abortions; Women with RPL in whom the causes of abortion were known were excluded by performing anatomical, hormonal, and chromosomal tests, and tests for infection (toxoplasmosis, cytomegalovirus, rubella, hepatitis B and C, AIDS). Women with RPL with autoimmune causes, including anti-cardiolipin antibody (ACLA), and lupus anticoagulant were also excluded from the study. |  | Ethnically matched normal controls with at least two successful pregnancies; Both patients and controls had each had a single partner during their reproductive age. |
| Quintero,2006^a^ [32] | Mexico | Caucasian | Women with at least three miscarriages |  | Healthy women |
| Zhou,2006^b^ [X] | China | Asian | Fertile women with three or more unexplained consecutive pregnancy losses with the same partner. |  | Healthy fertile women from Han population |
| Zammiti,2009 [33] | Tunisia | Caucasian | Fertile women with three to six unexplained consecutive pregnancy losses with the same partner. Chromosomal aberrations and Rh incompatibility were ruled out before inclusion in the study. Exclusion criteria included preclinical miscarriages/biochemical pregnancy. In addition, endocrine disorders including diabetes mellitus [glycated hemoglobin (HbA1c) levels R6.00], autoimmune disease, arterial hypertension, liver function abnormalities, drug abuse, previously known systemic disease, and personal and/or family history of thromboembolism were excluded. Additional exclusion criteria included abnormal thyroid function, thyroid antibodies. |  | Control subjects were matched with patients according to age (P¼.703), and a number of risk factors (smoking, alcohol consumption, oral contraceptive use) were similar to those of the RM cases. |
| Liu,2010 [34] | China | Asian | Inclusion criteria were defined as continuous two or more times of the spontaneous loss of pregnancy prior to the 22nd gestational week of pregnancy. All recruited people were Han population. |  | Controls were individuals of proven fertility, with normal menstrual cycles and ovary morphology, without the history of subfertility treatment. |
| Palmirotta,2010 [35] | Italy | Caucasian | The following tests were performed to exclude known causes of abortion or infertility: hysteroscopy, serial ultrasound, parental karyotypes, hormonal tests, and tests for infection (toxoplasmosis, cytomegalovirus, rubella, hepatitis B and C, AIDS). Thrombophilic conditions were not tested for prior to enrolment and were not used as inclusion or exclusion criteria, but women with autoimmune causes, including anticardiolipin antibody and lupus anticoagulant (LA), were excluded from the study. |  | One hundred unrelated healthy fertile women (mean age 38 + 5 years, ranging from 23 to 48) from the same geographical area as patients were evaluated. |
| Gupta,2012 [36] | India | Asian | All the patients experiencing repeated early pregnancy losses were screened for various known causes of miscarriages including, parental chromosomes, day two hormone levels of Follicle Stimulating Hormone (3–11 U/L), Leutinizing Hormone (3–12 U/L) and testosterone (0.5–3 nmol/L), antiphospholipid antibodies including lupus anticoagulant (PLR, 0.8–1.05) and anticardiolipin antibodies (IgG 0–12GPL units, IgM 0–5 MPL units), and prothrombotic risk factors including activated protein-C resistance (APCR, 2.6–4.36 ratio), factor V Leidden and prothrombin mutations, investigation of luteal phase insufﬁciency, prolactin dosage, glycaemic curve, thyroid hormone levels, investigation of Toxoplasmosis, Cytomegalovirus, Rubella, HIV, group B Streptococci, Chlamydia trachomatis, hepatitis B and C and bacterial vaginosis. The uterine cavity was investigated for cervical incompetence by hysteroscopy, hysterosalpingography, and serial ultrasound. |  | The control group consisted of 500 healthy parous women of the same ethnicity as that of RM patients [14] with at least two live births with no history of miscarriages, preeclampsia, ectopic pregnancy or preterm delivery as well as systemic diseases. |
| Alkhuriji,2013 [37] | Saudi Arabia | Caucasian | Routine analysis at the hospital laboratory were performed to exclude known causes of abortion: parental karyotypes; hormone levels; toxoplasmosis; cytomegalovirus; rubella; antiphospholipid antibodies; protein C; protein S; glucose level; hysteroscopy; hysterosalpingography; and serial ultrasound when needed. The criteria for inclusion  were: females presenting with unexplained RSA after all the tests mentioned above were normal. |  | The reference population (controls) consisted of 65 women who had at least 2 children, and were without known pregnancy losses or any known medical illnesses. |
| Lee,2013 [38] | South Korea | Asian | None of the patients had a history of smoking or alcohol use. Patients with recurrent pregnancy losses due to anatomic, hormonal, chromosomal, infectious, autoimmune, or thrombotic causes were excluded from the study group. |  | The women in the control group were recruited from medical center and met the following enrollment criteria: regular menstrual cycles, a history of at least 1 naturally conceived pregnancy, no history of pregnancy loss, and karyotype 46, XX. |
